# Sex-based differences in association between circulating T cell subsets and disease activity in untreated early rheumatoid arthritis patients

**DOI:** 10.1186/s13075-018-1648-2

**Published:** 2018-07-20

**Authors:** Jonathan Aldridge, Jayesh M. Pandya, Linda Meurs, Kerstin Andersson, Inger Nordström, Elke Theander, Anna-Carin Lundell, Anna Rudin

**Affiliations:** 1 Department of Rheumatology and Inflammation Research, Institute of Medicine, Sahlgrenska Academy of University of Gothenburg, Box 480, S-405 30 Gothenburg, Sweden; 2 Department of Rheumatology, Skåne University Hospital Lund and Malmö, Lund University, Lund, Sweden

**Keywords:** T cells, Rheumatoid arthritis, Disease activity, Sex

## Abstract

**Background:**

It is not known if sex-based disparities in immunological factors contribute to the disease process in rheumatoid arthritis (RA). Hence, we examined whether circulating T cell subset proportions and their association with disease activity differed in male and female patients with untreated early rheumatoid arthritis (ueRA).

**Methods:**

Proportions of T cell subsets were analyzed in peripheral blood from 72 ueRA DMARD- and corticosteroid-naïve patients (50 females and 22 males) and in 31 healthy age- and sex-matched controls. Broad analysis of helper and regulatory CD4^+^ T cell subsets was done using flow cytometry. Disease activity in patients was assessed using DAS28, CDAI, swollen joint counts, tender joint counts, CRP, and ESR.

**Results:**

Multivariate factor analyses showed that male and female ueRA patients display distinct profiles of association between disease activity and circulating T cell subset proportions. In male, but not female, ueRA patients Th2 cells showed a positive association with disease activity and correlated significantly with DAS28-ESR, CDAI, and swollen and tender joint counts. Likewise, proportions of non-regulatory CTLA-4^+^ T cells associated positively with disease activity in male patients only, and correlated with DAS28-ESR. In contrast, there was a negative relation between Th1Th17 subset proportions and disease activity in males only. The proportions of Th17 cells correlated positively with DAS28-ESR in males only, while proportions of Th1 cells showed no relation to disease activity in either sex. There were no significant differences in proportions of T cell subsets between the sexes in patients with ueRA.

**Conclusions:**

Our findings show sex-based differences in the association between T cell subsets and disease activity in ueRA patients, and that Th2 helper T cells may have a role in regulating disease activity in male patients.

**Electronic supplementary material:**

The online version of this article (10.1186/s13075-018-1648-2) contains supplementary material, which is available to authorized users.

## Background

Rheumatoid arthritis (RA) is a chronic and systemic inflammatory disease characterized by synovial inflammation and progressive destruction of joint cartilage and bones [1]. Genetic association studies strongly support the role of CD4^+^ T cells in promoting RA pathology. So far, alleles in the human leukocyte antigen (HLA)-DRB1 region, known as the shared epitope, display the strongest genetic association with RA [2]. Additional genetic loci have also been found to be linked with RA, among them many contain genes linked to T cell activation and function (reviewed in [3]). Still, identification of specific CD4^+^ T cell phenotypes or functions that are most relevant in this disease, particularly in early and preclinical RA, has been challenging. Traditionally, RA has been thought to be a Th1- and Th17-mediated disorder [1]. However, by combining single-cell analysis and next-generation sequencing in TCR repertoire analysis, it was shown that a majority of the most expanded CD4^+^ T cell clones from patients with established RA, both in synovial fluid and in peripheral blood, expressed phenotypes other than the Th1 or Th17 subsets [4]. Furthermore, synovial CXCR5^neg^PD-1^hi^ T peripheral helper (Tph) cells has recently been implicated as contributors in established RA [5]. In a cohort of untreated early RA (ueRA) patients, we also recently demonstrated that the balance of helper T cell subsets in blood of ueRA patients is skewed towards Th2 cells relative to healthy controls [6].

RA has been shown to be a sexually dimorphic condition with current data suggesting that prevalence, disease course, and treatment outcome varies between men and women. The prevalence of RA is approximately threefold higher in females than in males with several observational studies also suggesting that women have a more detrimental disease course than men [7, 8]. For example, in an 8-year prospective follow-up of an early RA study, women had more disability than men despite similar medication [9]. Male RA patients have also shown a higher remission rate than female patients in response to biologic and non-biologic disease-modifying anti-rheumatic drug (DMARD) treatments [7, 8]. However, another study showed that males were only more likely to achieve sustained remission in early RA, not in established RA, when treated with both biologic and non-biologic DMARD [10]. These results indicate that the initial immunological mechanisms responsible for RA may differ in males and females.

There are several disparities between men and women that have been shown to affect both innate and adaptive immune function [11]. Sex hormones are one such contributor, known to affect the immune system via their effects on multiple immune cell subsets as well as on stromal cells [11, 12]. As of yet, results from immunological studies of male and female patients with RA have not been reported separately, except for a recent study where we reported that levels of pro-inflammatory chemokines in blood were higher in ueRA patients than in healthy control (HC) subjects [13]. In this study, there was a positive association between eotaxin levels and disease activity in male patients, while these variables were inversely related among females. When male and female RA patients are reported as a single cohort in studies of immunopathogenesis and biomarkers there is risk that associated data are masked by opposing results by the two sexes. Hence, there is a need to examine both immunological components and their relation to disease activity measures separately in male and female patients with RA. Furthermore, sex-based differences need to be evaluated in a group of DMARD-naïve early RA patients since treatment with biologic and non-biologic DMARD alter the profile of both immune cells and soluble inflammatory mediators [14, 15].

To address these gaps in knowledge, we here investigated sex-based differences in the association between T cell subset proportions in peripheral blood and disease activity in ueRA patients. Using a broad-scale analysis of T cell subsets based on chemokine receptor expression, and relating these to disease activity measures, we demonstrate differential profiles of association between T cell subsets and disease activity in male and female ueRA patients.

## Methods

### Patients and healthy controls

The patient group comprised of 72 treatment-naïve subjects who were newly diagnosed with rheumatoid arthritis (RA) according to the American College of Rheumatology (ACR)/European League Against Rheumatism (EULAR) 2010 criteria. The inclusion criteria were as follows: ≥18 years old, ≥ 2 swollen joints and ≥ 2 tender joints (based on 66/68 joint count), rheumatoid factor (RF)-positive or anti-citrullinated protein antibodies (ACPA)-positive or C-reactive protein (CRP) ≥10 mg/L, at least moderate disease activity (> 3.2) by composite index disease activity score in 28 joints (DAS28-CRP), symptom duration < 24 months (retrospective patient-reported symptoms), and no treatment with any DMARD or corticosteroids. Blood samples were drawn from the DMARD- and corticosteroid-naïve patients within 1–2 weeks after RA diagnosis. The patient group was compared to a group of 31 age- and sex-matched HC subjects. Characteristics of female and male ueRA patients and HC are shown in Table 1. The median age of the whole patient cohort (59 years, range 21–80 years) and the HC cohort (55 years, range 20–75 years) was not significantly different (*P* = 0.47). Neither was the median age of ueRA female patients and ueRA male patients significantly different (Table 1). In addition, there were no significant differences between the median age of ueRA female patients and HC female subjects (*P* = 0.81), between the median age of ueRA male patients and HC male subjects (*P* = 0.38), or between the median age of HC female subjects and HC male subjects (Table 1). Furthermore, there was no significant difference between male and female ueRA patients with regard to disease activity measures, or proportions of patients positive for ACPA or RF (Table 1). The patients were recruited at the Rheumatology Clinics at Sahlgrenska University Hospital and at Skåne University Hospital, Sweden. All samples were analyzed at the Clinical Immunology Laboratory at the Sahlgrenska University Hospital in Gothenburg by the same staff to minimize variability. The study was approved by the regional ethics committees of Gothenburg and Lund, Sweden, and all patients signed an informed consent form.

**Table 1:** Baseline characteristics of female and male early diagnosed untreated RA patients and healthy controls

	ueRA female (*n* = 50)	ueRA male (*n* = 22)	*P* value	HC female (*n* = 18)	HC male (*n* = 13)
Age, yr.^*a*^	60.5 (21–78)	56 (28–80)	0.87^c^	56 (20–72)	55 (27–75)^e^
Symptom duration, months^*a,b*^	6 (2–23)	3.9 (1–21)	0.07^c^		
CRP, mg/L^*a*^	9 (0.3–180)	10 (2–113)	0.29^c^		
ESR, mm/hour^*a*^	28 (5–120)	22.5 (1–85)	0.33^c^		
SJC66^*a*^	11.5 (3–30)	9 (2–18)	0.08^c^		
TJC68^*a*^	13 (2–47)	15.5 (3–26)	0.79^c^		
SJC28^*a*^	9 (2–24)	7 (2–15)	0.15^c^		
TJC28^*a*^	8.5 (0–27)	9 (1–16)	0.93^c^		
DAS28-CRP^*a*^	4.9 (2.7–8.3)	5.2 (3.4–6.4)	0.50^c^		
DAS28-ESR^*a*^	5.3 (2.6–8.7)	5.5 (2.9–6.7)	0.83^c^		
CDAI^*a*^	28.3 (10.1–68.7)	28.0 (10.5–40.6)	0.40^c^		
ACPA+, *n* (%)^f^	42 (84)	17 (77)	0.52^d^		
RF+, *n* (%)^g^	38 (76)	14 (64)	0.40^d^		
ACPA+ and RF+, *n* (%)^f,g^	35 (70)	13 (59)	0.42^d^		
ACPA- and RF-, *n* (%)^f,g^	5 (10)	4 (18)	0.44^d^		
Smoker (%)^h^	8 (17)	3 (14)	> 0.99^d^		

*ACPA* anti-citrullinated protein/peptide antibodies, *CDAI* clinical disease activity index, *CRP* C-reactive protein, *DAS28* disease activity score in 28 joints, *ESR* erythrocyte sedimentation rate, *HC* healthy controls, *RF* rheumatoid factor, *SJC 28/66* swollen joint counts of 28/66, *TJC 28/68* tender joint counts of 28/68, *ueRA* untreated early rheumatoid arthritis

^a^Median and range

^b^Retrospective patient-reported pain in the joints before RA diagnosis

^c^Difference between ueRA female patients and ueRA male patients, Mann-Whitney *U* test

^d^Difference between ueRA female patients and ueRA male patients, Fisher’s exact test

^e^Difference between HC female age and HC male age, *P* = 0.53, Mann-Whitney *U* test

^f^Patients with ACPA levels ≥20 IU/ml are considered ACPA+

^g^Patients with RF levels ≥20 IU/ml are considered RF+

^h^Current daily smoker (data available in n_female_ = 47, n_male_ = 22)

### Clinical evaluation

Evaluation of disease activity in patients was done by assessing the following parameters: Swollen Joint Counts of 66 joints (SJC 66), Tender Joint Counts of 68 joints (TJC 68), Swollen Joint Counts in 28 joints status (SJC 28), Tender Joint Counts in 28 joints status (TJC 28), CRP, erythrocyte sedimentation rate (ESR), DAS28 [16], and Clinical Disease Activity Index (CDAI) [17]. ACPA positivity was determined by multiplexed anti-CCP test (BioPlex from BioRad, Hercules, CA, USA) and RF positivity was determined by nephelometry (Beckman Coulter, Brea, CA, USA). Patients with ≥20 IU/ml anti-CCP antibodies or RF in serum were considered ACPA- or RF-positive, respectively.

### Definition, analysis and characterization of T cell subsets

**Fig. 1:**
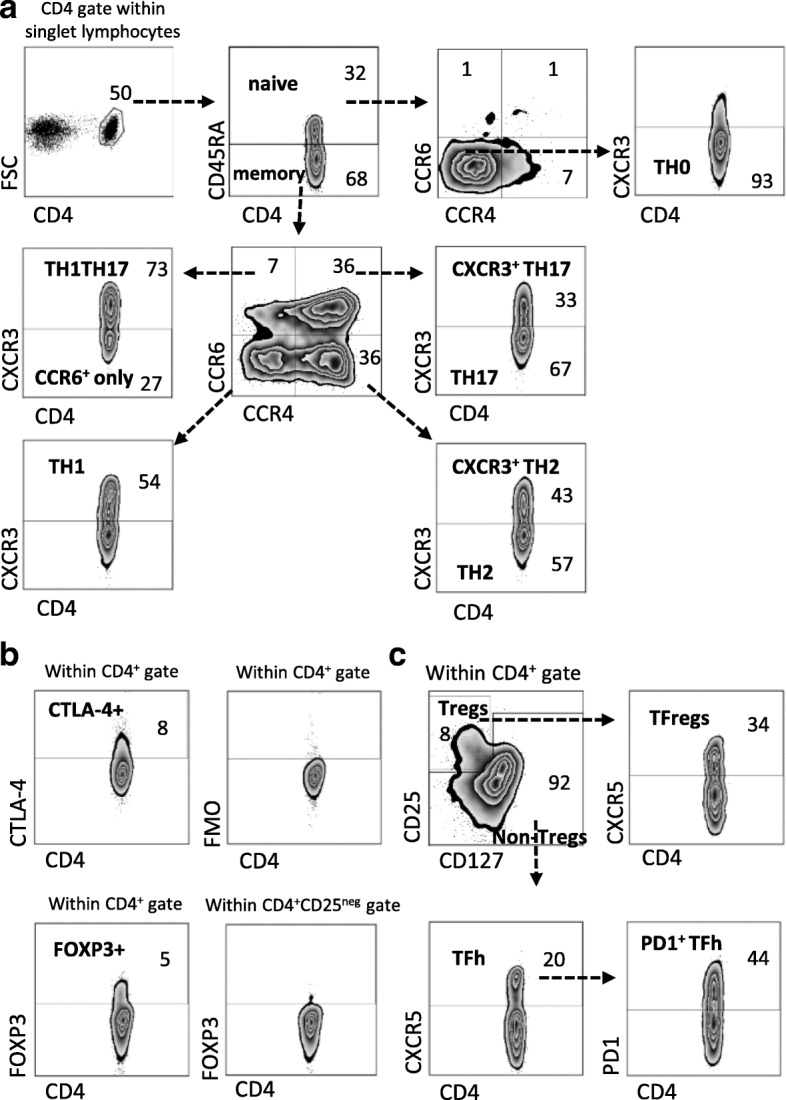
Gating strategy of CD4+ T cell subsets. The gating strategy (gating result from a representative female RA patient) was as follows: (**a**) singlet PBMCs were gated for lymphocytes and then further gated for CD4^+^ T cells. CD4^+^ cells where then divided into naïve (CD45RA^+^) and memory (CD45RA^neg^) subsets. From naïve cells, CCR4^neg^CCR6^neg^CXCR3^neg^ cells were defined as Th0. Memory cells were divided into four subsets based on CCR4 and CCR6 expression, each of which was the further divided based on CXCR3 expression; Th1 (CCR4^neg^CCR6^neg^CXCR3^+^), Th2 (CCR4^+^CCR6^neg^CXCR3^neg^), CXCR3^+^Th2 (CCR4^+^CCR6^neg^CXCR3^+^), Th17 (CCR4^+^CCR6^+^CXCR3^neg^), CXCR3^+^Th17 (CCR4^+^CCR6^+^CXCR3^+^), Th1Th17 (CCR4^neg^CCR6^+^CXCR3^+^), and CCR6^+^ only (CCR4^neg^CCR6^+^CXCR3^neg^). (**b**) The cutoff for CTLA-4 positivity on CD4^+^ T cells were determined using fluorescence minus one (FMO) and cutoff for FOXP3 positivity in CD4^+^ cells was based on FOXP3 expression in CD25^neg^ gated CD4^+^ cells. (**c**) Regulatory T cells (Tregs) were defined by CD25^+^CD127^low^ expression, while the remaining cells were defined as non-Tregs. CXCR5^+^ Tregs were defined as follicular regulatory T cells (TFregs) and CXCR5^+^ non-Tregs as follicular helper T cells (TFh).

Peripheral blood mononuclear cells (PBMCs) were separated from whole blood (sampled from patients within 1–2 weeks after RA diagnosis) using Lymphoprep (Axis-Shield, Oslo, Norway). Small aliquots of fresh blood were used for cell counts (True count, TC) using BD TruCOUNT Absolute Counting Tubes with addition of CD45 PerCP and CD4 APC-H7 antibodies (BD Biosciences, San Jose, CA, USA). In isolated fresh PBMCs, T cell subsets were defined and analyzed using flow cytometry, as previously described in detail [6]. In brief, without any ex vivo stimulations, PBMCs were stained with fluorochrome-conjugated monoclonal antibodies against the following molecules: CD4, CD45RA, CCR4, CCR6, CXCR3, CXCR5, CD127, PD-1, and CD25, and to evaluate FOXP3^+^ and CTLA-4^+^ cells, intracellular staining was performed (full list of antibodies available in Additional file 1: Table S1) [6]. Stained samples were acquired by the use of FACSCanto II (BD Biosciences) equipped with FACS Diva software (BD Biosciences). Flow cytometry data was analyzed in FlowJo software (Tree Star, Ashland, OR, USA). T helper subsets were defined by surface chemokine receptor expression. The gating strategy to define different T cell subsets is previously described in [6] and also presented in Fig. 1. The phenotypes of defined T cell subsets were confirmed by lineage specifying transcription factor expression analysis by qPCR and cytokine secretion analysis by Cytometric Bead Array (BD Biosciences) as previously shown [6].

### Statistical analysis

Multivariate factor analysis (SIMCA-P+ software; Umetrics, Umeå, Sweden) was used to analyze T cell and disease activity data. Principal component analysis (PCA) was performed in order to evaluate association between proportions of T cell subsets and disease activity measures. Linear OPLS models were implemented to examine sex-based differences in the associations of T cell subsets with disease activity measures. Orthogonal projection to latent structures discriminant analysis (OPLS-DA) was implemented to investigate sex-based differences in T cell subset proportions in ueRA and HC. The two-class discrimination OPLS-DA model has one predictive component and one orthogonal. Log transformation was used to normalize data and further scaled to unit variance in the SIMCA software by dividing each variable by 1/standard deviation (SD), so that all the variables were given equal weight regardless of their absolute value. The scale presented on the axes of the PCA and OPLS plots is a dimensionless scale, and the loading vector is normalized to length one. The quality of the OPLS models was assessed based on the parameters R2 (i.e. how well the variation of the variables is explained by the model), and Q2, (i.e. how well a variable can be predicted by the model). In order to avoid mass significance, univariate analysis was performed exclusively on the variables that contributed most to the respective OPLS models. Univariate analyses were performed using two-tailed Mann-Whitney *U* test and two-tailed Spearman’s Rank-Order Correlation (GraphPad Prism Software, La Jolla, CA, USA) as described in the figure legends. The strength of correlation was determined based on Spearman’s rank correlation coefficient (r) values (*r* = 0.2–0.39 weak correlation, *r* = 0.4–0.59 moderate correlation, *r* = 0.6–0.79 strong correlation, *r* > 0.8 very strong correlation). A *P* value ≤0.05 was regarded as being statistically significant (*P ≤ 0.05, **P ≤ 0.01, ***P ≤ 0.001 and ****P ≤ 0.0001).

## Results

### Male and female untreated early RA patients show different profiles of association between T cell subsets and disease activity

**Fig. 2:**
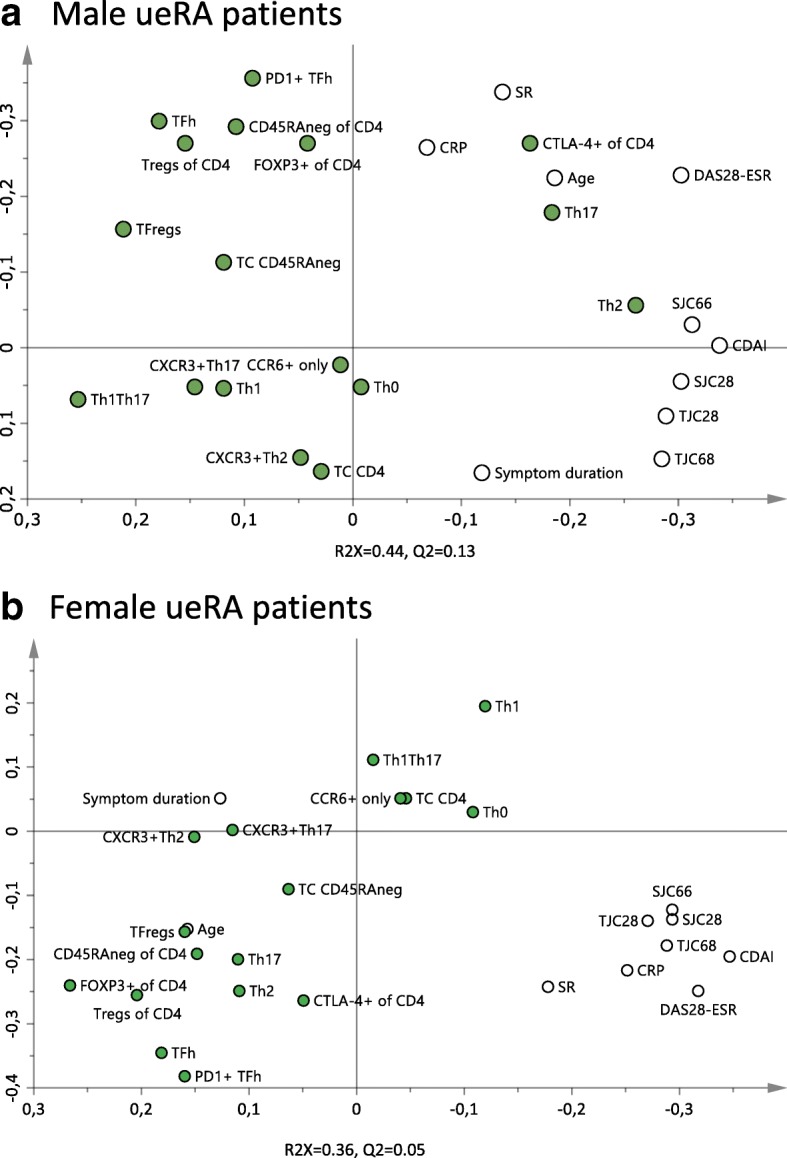
Relationship between T cell subset proportions and disease activity measures in male and female untreated early RA patients. Principal component analysis (PCA) plots depicting the association between CD4^+^ T cell subsets proportions and disease activity measures (**a**) in male (*n* = 20–22) and (**b**) in female (*n* = 48–50) ueRA patients. Variables projected close to each other on the same side of the axis associate positively, while variables on opposite sides of an axis associate negatively. *Filled symbols* indicate CD4^+^ T cell subset variables and *open symbols* indicate disease activity variables

Based on the gating strategy presented in Fig. 1a-c we first examined the proportions of T helper (Th) and regulatory CD4^+^ T cell subsets in male and female ueRA patients. By the use of cluster analysis, i.e. PCA, we next investigated whether patient sex affects the relationship between proportions of T cell subsets and common disease activity measures used when assessing RA patients (Fig. 2a-b). In both sexes, all disease activity variables were projected in the two right hand quadrants. However, while the Th2, Th17, and CTLA-4^+^ T cell subset proportions associated positively with several disease activity measures in males (Fig. 2a), none of the T cell subsets were projected in the same quadrant as that of disease activity variables among females (Fig. 2b). Thus, results indicate that the association between T cell subset proportions and disease activity differ in male and female ueRA patients.

### Th2 helper T cells correlate with increased disease activity in male but not in female ueRA patients

**Fig. 3:**
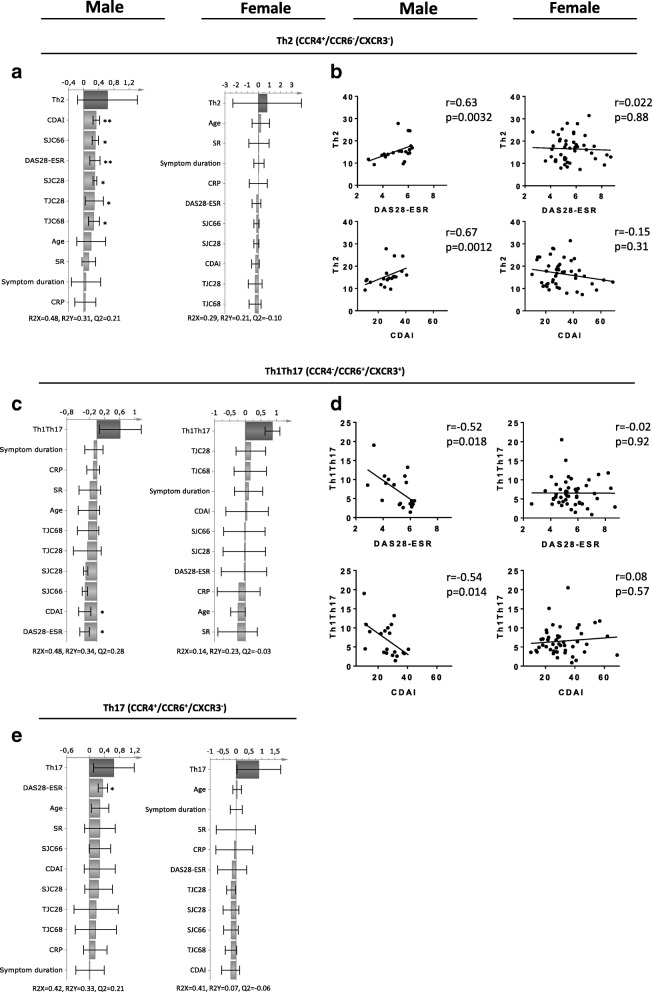
Relationship between Th2, Th1Th17, and Th17 subset proportions and disease activity measures in male and female untreated early RA patients. Multivariate factor analysis was performed to investigate sex differences in the association of Th2, Th1Th17, and Th17 T cell subset proportions with multiple disease activity measures in ueRA patients. OPLS column loading plots depicting the association between (**a**) Th2, (**c**) Th17Th17 and (**e**) Th17 subset proportions (Y-variables) and disease activity measures (X-variables) in male (*n* = 20) and female (*n* = 48) ueRA patients. In OPLS column loading plot, X-variables represented by *bars pointing in the same direction* as Y-variable are positively associated with Y-variable, whereas X-variables with *bars pointing in the opposite direction* are inversely related to the Y-variable. Correlation analyses between the proportion of (**b**) Th2 or (**d**) Th1Th17 cells with disease activity measures in male and female ueRA patients, respectively. *P ≤ 0.05 and **P ≤ 0.01 (Spearman’s rank correlation test). Regression lines are presented in the correlation plots

Having observed sex-based differences in association between T cell subset proportions and disease activity in the PCA analysis, we examined the association of individual helper T cell subsets with multiple disease activity measures, symptom duration, and age using multivariate OPLS analysis. In males, we found that proportions of Th2 cells displayed a positive association with several disease activity measures (Fig. 3a), and correlated significantly with DAS28-ESR, CDAI (Fig. 3b), swollen joint counts (SJC28 *r* = 0.45 *P* = 0.05 and SJC66: *r* = 0.48 *P* = 0.03) and tender joint counts (TJC28 *r* = 0.59 *P* = 0.007 and TJC68: *r* = 0.54 *P* = 0.01). In female ueRA patients, no relation was observed between proportions of Th2 cells and disease activity measures (Fig. 3a-b). In contrast to Th2, proportions of Th1Th17 cells displayed a negative association and inverse correlation with disease activity measures in male patients, while no relation was observed in females (Fig. 3c-d). The Th17 cells did show patterns of positive association with disease activity in male patients, while females displayed a pattern of negative association (Fig. 3e). However, significant univariate correlation between disease activity measures and proportions of Th17 cells was only observed in male patients (DAS28-ESR, *r* = 0.47 *P* = 0.035). The CXCR3^+^ Th17 cell proportions in female patients showed significant inverse correlation with swollen joint counts (SJC28, *r* = − 0.41 *P* = 0.004 and SJC66, *r* = − 0.38 *P* = 0.007).

Although the median level of disease activity was similar in men and women with ueRA, higher disease activity was found in a fraction of the female patients compared to males, which could be a confounding factor for the results. However, exclusion of the female patients with disease activity above the maximum value observed in male patients showed little to no effect to the r and *P* values in the correlation analyses (relation between Th2 and disease activity before exclusion: *r* = 0.022 and *P* = 0.88 vs after exclusion: *r* = 0.097 and *P* = 0.54). In an attempt to demonstrate that the number of male patients was sufficiently high, additional analyses were performed in which the number of female patients was randomly reduced to 20 (equal to the number of male patients). Univariate analyses were then performed as described previously, investigating the relation between proportions T cell subsets and disease activity. Reduction in the number of females did not have a significant impact on the relation between T cell subset proportions and disease activity (e.g. relation between Th2 and disease activity before reduction: r = 0.022 and *P* = 0.88 vs after reduction: *r* = − 0.082 and *P* = 0.73). In summary, these findings suggest significant sex-based differences in the contribution of Th2, Th1Th17, and CXCR3^+^ Th17 cells to disease severity in RA.

### CTLA-4^+^ conventional CD4^+^ T cell subsets correlate with disease activity in male but not in female ueRA patients

**Fig. 4:**
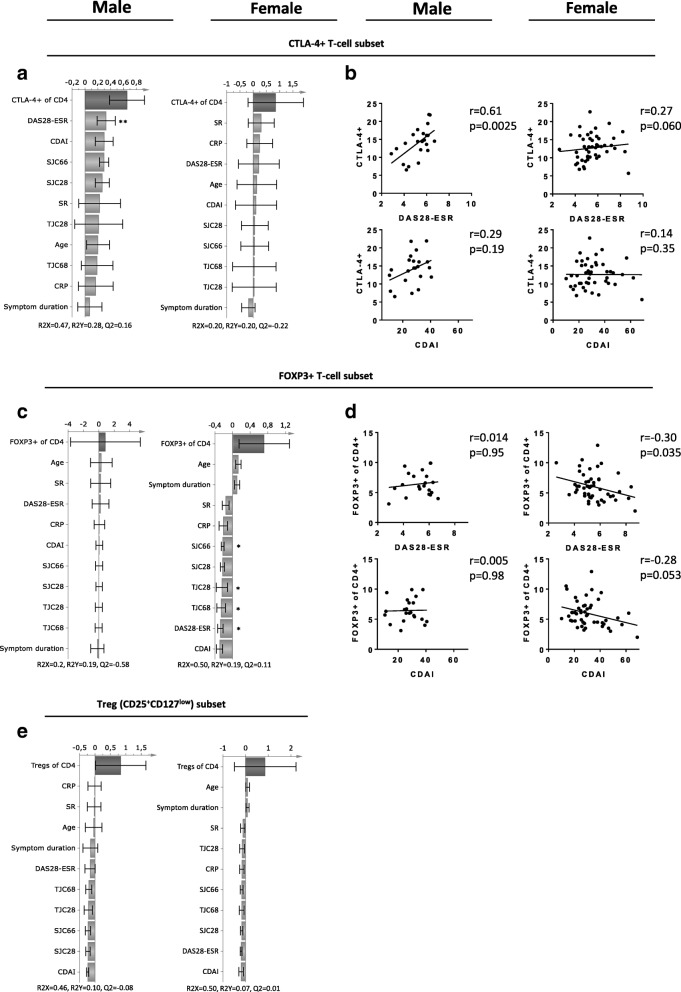
Relationship between CTLA-4+, FOXP3+ and regulatory T cell subset proportions and disease activity measures in untreated early RA patients. Multivariate factor analysis was performed to investigate sex-based differences in the associations of CTLA-4^+^, FOXP3^+^, and regulatory T cell subset proportions with multiple disease activity measures in ueRA patients. OPLS column loading plots depicting the association between (**a**) CTLA-4^+^, (**c**) FOXP3^+^, and (**e**) regulatory T cell subset proportions (Y-variables) and disease activity measures (X-variables) in male (*n* = 22) and female (n = 48–50) ueRA patients. Correlation analyses between the proportion of (**b**) CTLA-4+ or (**d**) FOXP3+ T cell subsets proportions with disease activity measures in male and female ueRA patients, respectively.*P ≤ 0.05 and **P ≤ 0.01 (Spearman’s rank correlation test). Regression lines are presented in the correlation plots

Next, we examined the association between proportions of CTLA-4^+^, FOXP3^+^ and regulatory T cell subsets and disease activity in male and female ueRA patients (Fig. 4a-e). We found that the CTLA-4^+^ T cell fractions displayed a profile of positive association with the majority of disease activity measures in male patients, but not in female patients (Fig. 4a). These results were also corroborated in univariate analysis as the proportions of CTLA-4^+^ T cells correlated positively with DAS28-ESR in males, while no significant correlations were observed in females (Fig. 4b). CTLA-4 is often used as a marker for regulatory T cells, but CTLA-4 is also expressed by activated conventional T cells. Upon further analyses, we found that both proportions of regulatory and non-regulatory CTLA-4^+^ T cells showed moderate to strong correlation with DAS28-ESR in males (*r* = 0.57 *P* = 0.006 and *r* = 0.62 *P* = 0.002, respectively). Male regulatory, but not non-regulatory, CTLA-4^+^ T cells also showed moderate correlation to CDAI, SJC28, SJC66 and TJC28 (*r* = 0.54 *P* = 0.01, *r* = 0.44 *P* = 0.04, r = 0.44 *P* = 0.039 and *r* = 0.51 *P* = 0.01, respectively). In female patients, only regulatory CTLA-4^+^ T cells showed a weak correlation to DAS28-ESR (*r* = 0.29 *P* = 0.05), whereas no significant correlation between the non-regulatory CTLA-4^+^ T cell population and disease activity was observed. In contrast to CTLA-4^+^ T cells, proportions of FOXP3^+^ T cells showed no association with disease activity measures in males, but a negative association in females (Fig. 4c). Univariate analysis confirmed a negative correlation between proportions of FOXP3^+^ T cells and DAS28-ESR (Fig. 4d), SJC66 (*r* = − 0.34 *P* = 0.02) and tender joint counts (TJC68: *r* = − 0.32 *P* = 0.03) in female patients. The proportions of regulatory T cells (CD25^+^CD127^low^CD4^+^ cells) displayed a weak pattern of negative association with disease activity measures in both sexes, but there were no significant correlations observed in either sex (Fig. 4e). Exclusion of the female patients with disease activity above the maximum value observed in male patients showed little to no effect to the r and *P* values for the correlation analyses (relation between CTLA-4+ and disease activity before exclusion: *r* = 0.27 and *P* = 0.060 vs after exclusion r = 0.29 and *P* = 0.062). Thus, although the proportions of CTLA-4^+^ T cells are elevated in blood of both male and female ueRA patients, this T cell subset correlates with disease activity in males only. Furthermore, non-regulatory CTLA-4^+^ T cells showed strong correlation to DAS28-ESR in males but not females.

### Male and female ueRA patients display a different profile of blood T cell subset proportions compared to their respective healthy controls

**Fig. 5:**
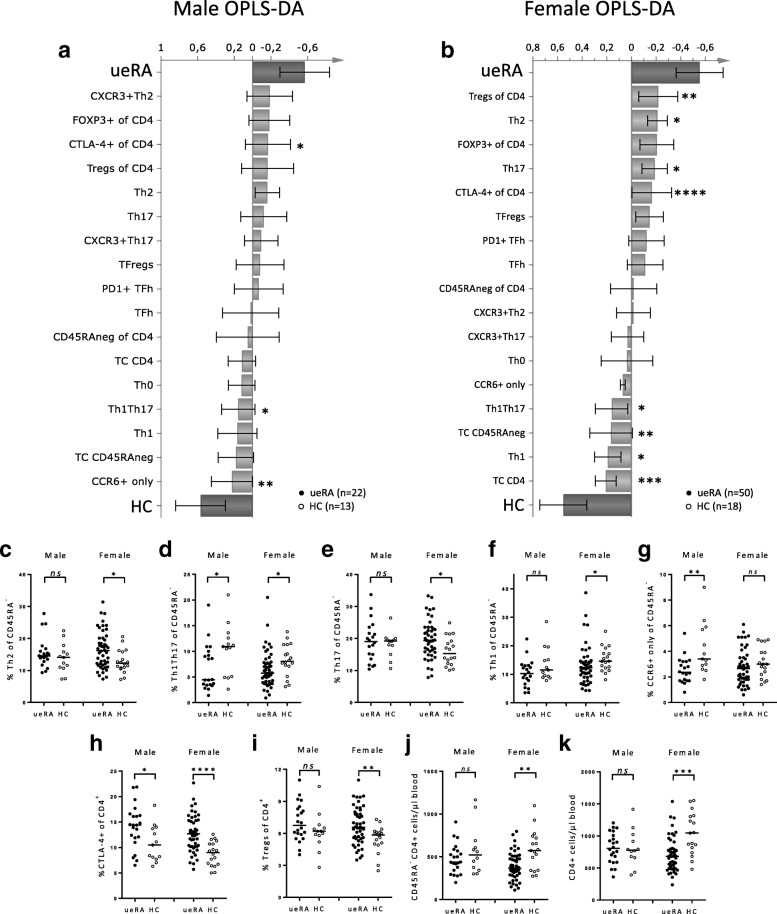
Effect of sex on the differential proportions of T cell subsets in untreated early RA patients compared to healthy controls. Multivariate factor analysis was performed to investigate whether the sex affects change in the profile of T cell subsets in ueRA compared to HC. OPLS-DA column loading plots showing the association between ueRA or HC (Y-variables) and T cell subset proportions (X-variables) (**a**) in males (n = 20–22) and (**b**) in females (*n* = 48–50). X-variables (*light grey bars*) pointing in the same direction as ueRA (*top dark grey bar*) are positively associated, whereas X-variables pointing in the opposite direction are positively associated with HC (*bottom dark grey bar*). Comparison of the proportions in CD45RA^neg^CD4^+^ cells of (**c**) Th2, (**d**) Th1Th17, (**e**) Th17, (**f**) Th1, (**g**) CCR6^+^ only, (**h**) CTLA-4^+^, (**i**) Tregs (CD25^+^CD127^low^), and counts of (**j**) CD45RA^neg^CD4^+^ (memory cells), and (**k**) all CD4^+^ cells between ueRA patients and HC are shown for males and females respectively. *Horizontal bars* indicate the median. Two-tailed Mann-Whitney *U* test. *P ≤ 0.05, **P ≤ 0.01, ***P ≤ 0.001 and ****P ≤ 0.0001

Previously, we have shown that ueRA patients display distinct profiles of T cell subset proportions compared to HC [6]. A similar multivariate distinctive pattern was observed in the present study (in a partially overlapping cohort) including a higher number of patients and healthy controls as shown in Additional file 1: Figure S1 [6, 13]. With a larger number of ueRA patients, we here investigated whether sex affects the differential T cell subset profile in ueRA patients in relation to age- and sex-matched HC. T cell subsets that showed the strongest association (positive or negative) with ueRA patients are identified in the OPLS-DA column plots in Fig. 5a and b for males and females respectively. In male patients, the differential profile of T cell subsets between ueRA and HC (Fig. 5a) was less pronounced than in females. Upon univariate analysis, the proportions of CTLA-4^+^ T cells were significantly higher in male ueRA patients (Fig. 5h), while the fractions of Th1Th17 and CCR6^+^ only cells were lower in ueRA patients relative to HC (Fig. 5d, g). In contrast, univariate analysis showed that female ueRA patients displayed a strong differential profile of T cell subset proportions compared to HC (Fig. 5b). Female patients had significantly higher proportions of Th2 and Th17 cells as well as elevated fractions of regulatory T cells (CD25^+^CD127^low^) and CTLA-4^+^ T cells compared to HC (Fig. 5c, e, h-i). Both the total count of CD4^+^ and CD45RA^neg^ T cells (Fig. 5j-k) as well as proportions of Th1Th17 and Th1 T cells were lower in female ueRA patients compared to female HC (Fig. 5d, f). Univariate analysis did not show any significant differences in T cell subset numbers or proportions between male and female patients with ueRA (Fig. 5c-k). Furthermore, no significant differences in T cell subset proportions between seropositive and seronegative patients was found in either males of females (data not shown).

In conclusion, these results show that there is a differential profile of T cell subset proportions in both male and female ueRA patients compared to their respective HC. Additionally, proportions of the CCR6^+^ only subset was shown to be higher in male HC than ueRA, which was not observed in females despite 2.3-fold higher number of female participants (Fig. 5g).

## Discussion

Sex-based differences in T cell subsets and their clinical relevance have not been explored, neither in early nor established RA. In this study, we found that male and female ueRA patients display distinct profiles of association between disease activity and certain T cell subsets. The proportions of Th2 cells associated positively with disease activity in male but not female ueRA patients, whereas Th1Th17 cells associated negatively with disease activity in male but not in female patients. Moreover, both sexes presented with increased proportions of CTLA-4^+^ T cells in ueRA, but proportions of these cells associated positively with disease activity in male ueRA patients only. To our knowledge, this is the first study demonstrating sex-based differences in the association between specific T cell subsets and disease activity measures in patients with RA.

The Th1 and Th17 cell subsets have been the target of RA research for many years and patients with established RA have indeed presented elevated frequency of citrulline-specific Th1 cells in circulation [18]. However, previous studies provide inconsistent results regarding the association of circulatory Th1 and Th17 cells with disease activity in RA [19–21]. Furthermore, a recent study showed that Th1 and Th17 cells make up only a small fraction of the most expanded CD4^+^ T cell clones from patients with established RA, both in peripheral blood and in the synovial fluid [4]. Few studies have explored the role of Th2 cells in RA pathogenesis, but it has been shown that synovial fluid in early arthritis patients who developed RA had elevated levels of the Th2 cytokine interleukin (IL)-4 [22]. Plasma levels of IL-4 and the Th2-related chemokine eotaxin have also been shown to be elevated in both male and female subjects with no prior symptoms of joint disease who later develop RA [23]. Additionally, we have previously reported that the proportion of Th2 cells in blood are increased in ueRA patients compared to HC in a combined cohort of males and females [6]. In the present study, when analyzing males and females separately, we found that only female ueRA patients displayed significantly elevated proportions of Th2 cells compared to HC. Moreover, we also observed sex-based disparities in the association between Th2 cells and disease activity. Male patients displayed a positive association pattern between Th2 cells and disease activity, while these factors were not related in females. This might explain why no association between Th2 cells and disease activity was previously found when analyzing the group of men and women together [6]. Supporting these sex-based differences, we have recently shown that blood plasma levels of eotaxin associated positively with disease activity in male ueRA patients, whereas it displayed negative association with disease activity in female ueRA patients [13]. Eotaxin, an eosinophil chemoattractant, is associated with Th2 immune response and allergic pulmonary diseases. Thus, our results point towards Th2 cells and eotaxin having a role in regulating the disease activity of male patients with early RA.

As discussed, previous studies provide inconsistent results regarding the association of Th1 and Th17 T cells and disease activity in RA. This may be due to that most such studies were performed on patients with variable disease duration, different treatment protocols, pooled cohorts of males and females, as well as analysis of T cells after ex vivo stimulation and culture. Thus, we here focused our investigation on a more homogenous patient group (untreated early RA) and analyzed fresh isolated PBMC without any intermediary culture or stimulation. We and others have shown that Th1Th17 cells display both Th1 and Th17 characteristics as they express both TBX21 and RORC and produce interferon (IFN)-γ and IL-17 [6, 24]. These cells, which express CXCR3 and CCR6, are also considered important to the disease process of RA [24, 25]. In this study, we show that proportions of circulating Th1Th17 cells displayed a negative association with clinical disease activity measures in male ueRA patients only. The proportion of circulatory Th17 cells was elevated compared to healthy controls in female patients but showed no significant correlation with disease activity in either sex. In contrast, previous studies have shown negative associations between circulating Th17 cells and disease activity measures in DMARD-naive RA patients with a mean disease duration of 10 and 21 months, respectively [26, 27]. However, the surface markers used to categorize the T cell subsets in these studies were different from ours. The first study defined Th17 cells using the markers CD4 and CD161, while the second study defined Th17 using CD4 combined with intracellular staining of IL-17. Owing to these disparities in defining the cell subsets, the results of the different studies are difficult to compare. Furthermore, sex-related disparities between T cell subsets and disease activity were not investigated in these studies.

We have recently reported that ueRA patients present with increased proportions of circulating CTLA-4 expressing CD4^+^ T cells compared to HC [6]. In the present study, we demonstrate that this is true for both sexes. However, when we investigated the association between CTLA-4^+^ T cells and disease activity, we found a positive association between CTLA-4^+^ T cells and disease activity in male ueRA patients only. Interestingly, RA susceptibility genes involved in T cell regulation (e.g. PTPN22) have also been found to have a stronger association with development of RA in males than in females [28–30]. CTLA-4 is expressed by the majority of regulatory T cells, but also by non-regulatory T cells, and functions as a negative regulator of T cell immune responses by competing with CD28 for binding to the costimulatory molecules CD80/CD86 on APCs [31]. These results suggest that the T cell inhibitory pathways linked to CTLA-4 may be particularly impaired in male ueRA patients, as immunosuppression is not achieved despite increased proportions of T cells expressing CTLA-4. In the present study, the FOXP3^+^ T cell subset displayed a clear profile of inverse association as well as negative correlations with disease activity measures in female patients. This suggests that at least in female early RA patients, FOXP3^+^ T cells may be able to regulate disease severity to some extent. In male patients, FOXP3^+^ T cells displayed no association with disease activity measures in multivariate analysis and no statistically significant correlations were found. This could be due to lower number of patients in the male cohort. Alternatively, male ueRA patients might have a higher degree of functional impairment in FOXP3^+^ regulatory T cells compared to female ueRA patients.

A possible explanation for the lack of positive associations between T cell subset proportions and disease activity in female patients could be that the immunological mechanisms driving early disease may be more heterogeneous in female RA patients compared to males. This could make identifying connections between immunological mechanisms and disease activity in females more difficult. It would also explain the more consistent response to treatment with methotrexate and tumor necrosis factor (TNF) inhibitors observed in males compared to females, even when controlling for baseline disease activity [8].

In our previous study, we observed a distinct profile of circulating T cell subset proportions in ueRA patients compared to HC [6], which was confirmed in the present study including a higher number of patients and healthy controls. When we separated groups into males and females, we found that female ueRA patients displayed a distinct T cell profile compare to HC. The same profile was not observed in male ueRA patients compared to HC, which could in part be due to the lower number of male relative to female patients in the study. However, in the analysis of T cell subsets in relation to disease activity the number of males was clearly sufficient.

Owing to the sexually dimorphic prevalence of RA as well the heterogeneous patient cohorts used in past studies, separation of the male and female patient cohorts and the use of DMARD-naïve early RA patients should be considered the first strength of this study. Secondly, by analyzing fresh PBMC without any ex vivo stimulation before analysis, we avoided the varying methods and duration of stimulation that may have led to inconsistent T cell data in prior studies. Lastly, by the use of multivariate factor analysis we reduced bias and multiplicity problems in the statistical analysis. However, only studying T cells in the circulation and not in the synovial fluid as well as having 2.3 times fewer male than female patients may be considered the major weaknesses.

## Conclusion

To the best of our knowledge, this is the first study evaluating sex-based differences in the association between T cell subsets and clinical disease activity measures in RA, more importantly in DMARD-naïve early RA patients. Results from our study point towards differential roles of certain T cell subsets in male and female patients in relation to disease activity in early RA. Specifically, implicating Th2 helper T cells as possible mediators in regulating disease activity of RA in males. Our results also demonstrate that male and female patients with early RA should be clinically evaluated separately and sex-specific differences should be taken into account in the development of immunomodulating therapies targeting RA.

## Additional file

Additional file 1: **Figure S1.** Differences in the proportions of T cell subsets between untreated early RA and healthy controls. OPLS-DA scatter and loading plots resulting from multivariate factor analysis of ueRA patients (male + female) vs HC (male + female). **Table S1.** The conjugate, clone, reference number and company of respective antibodies used for flow cytometry analysis. List of antibodies used in flow cytometry analysis and the respective conjugate, clone, reference number and company for each of these. (PPTX 99 kb)

## Abbreviations

ACPA: anti-citrullinated protein/peptide antibodies
CDAI: clinical disease activity index
CRP: C-reactive protein
DAS28: disease activity score in 28 joints
DMARD: disease-modifying anti-rheumatic drugs
ESR: erythrocyte sedimentation rate
HC: healthy controls
HLA: human leukocyte antigen
IL: interleukin
OPLS-DA: orthogonal projection to latent structures discriminant analysis
PBMC: peripheral blood mononuclear cells
PCA: principal component analysis
RA: rheumatoid arthritis
RF: rheumatoid factor
SJC 28/66: swollen joint counts of 28/66
TFh: T follicular helper
Th: T helper
TJC 28/68: tender joint counts of 28/68
Tph: T peripheral helper
Tregs: regulatory T cells
ueRA: untreated early rheumatoid arthritis
