# Supplementary material for: Sex-based differences in association between circulating T cell subsets and disease activity in untreated early rheumatoid arthritis patients
Source: Arthritis Res Ther. 2018 Jul 20;20:150. doi: 10.1186/s13075-018-1648-2 (PMC6053769; doi:10.1186/s13075-018-1648-2)

## Slide 1
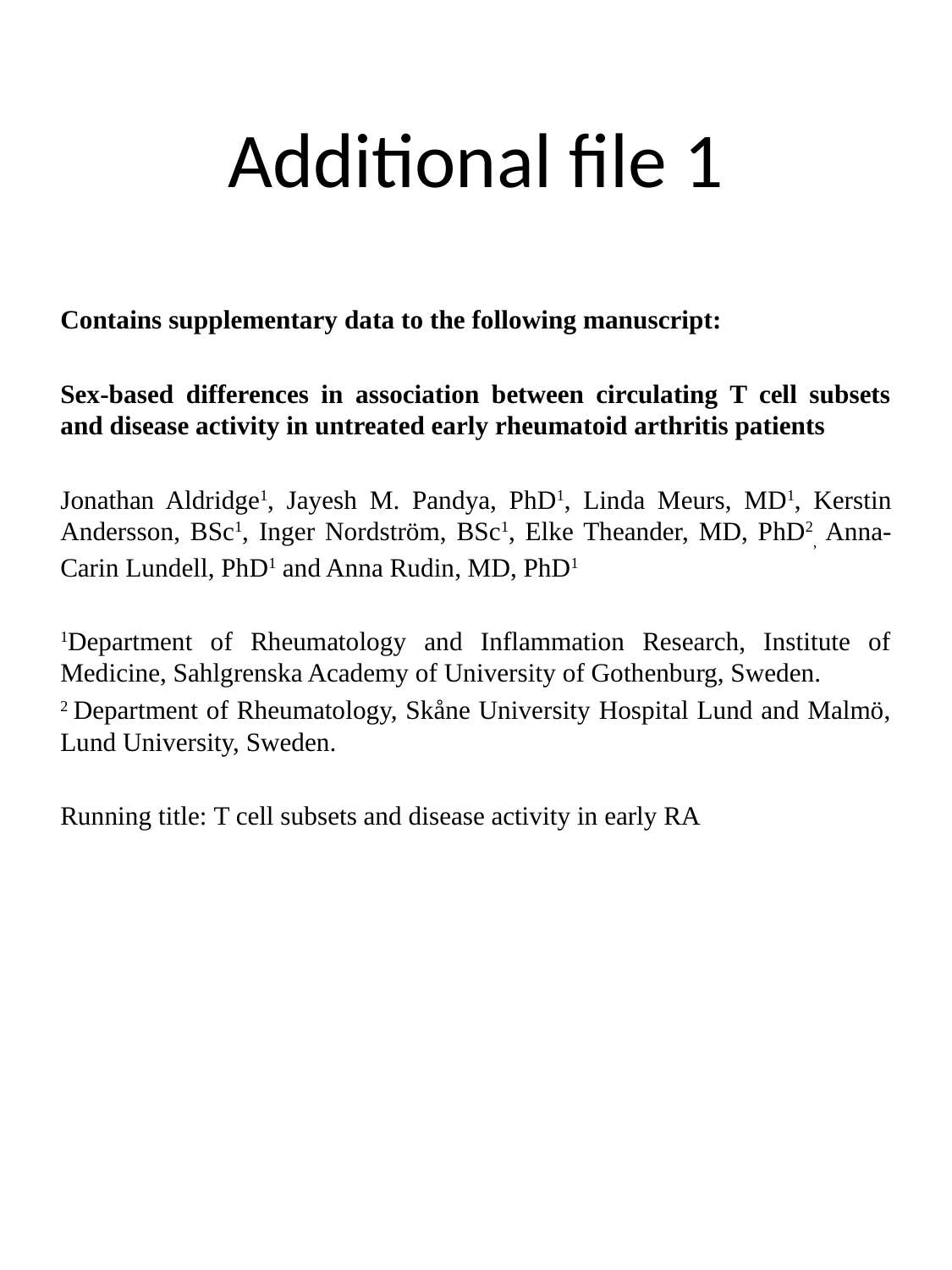

# Additional file 1
Contains supplementary data to the following manuscript:
Sex-based differences in association between circulating T cell subsets and disease activity in untreated early rheumatoid arthritis patients
Jonathan Aldridge1, Jayesh M. Pandya, PhD1, Linda Meurs, MD1, Kerstin Andersson, BSc1, Inger Nordström, BSc1, Elke Theander, MD, PhD2, Anna-Carin Lundell, PhD1 and Anna Rudin, MD, PhD1
1Department of Rheumatology and Inflammation Research, Institute of Medicine, Sahlgrenska Academy of University of Gothenburg, Sweden.
2 Department of Rheumatology, Skåne University Hospital Lund and Malmö, Lund University, Sweden.
Running title: T cell subsets and disease activity in early RA

## Slide 2
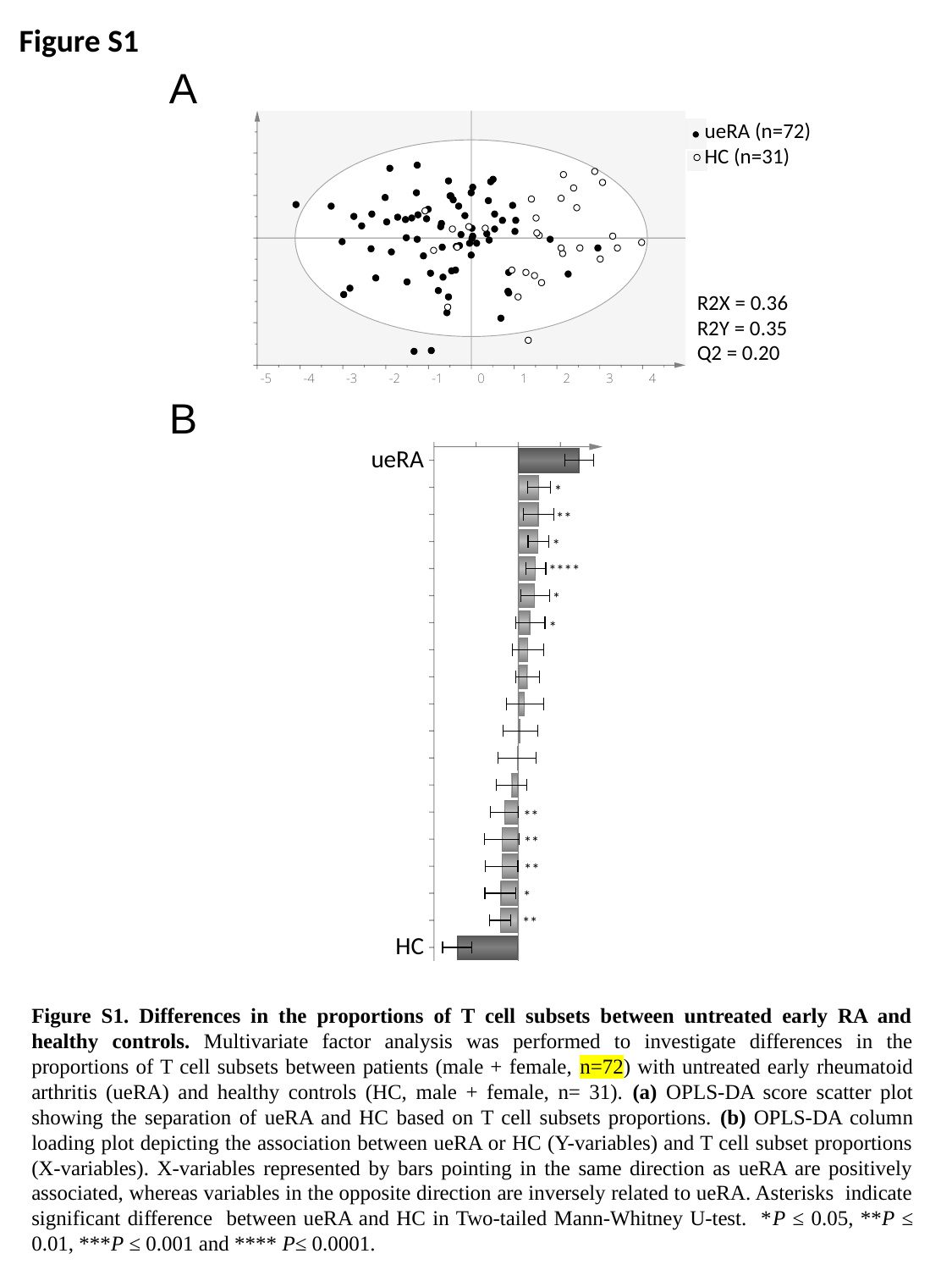

Figure S1
A
ueRA (n=72)
HC (n=31)
R2X = 0.36
R2Y = 0.35
Q2 = 0.20
B
ueRA
*
**
*
****
*
*
**
**
**
*
**
HC
Figure S1. Differences in the proportions of T cell subsets between untreated early RA and healthy controls. Multivariate factor analysis was performed to investigate differences in the proportions of T cell subsets between patients (male + female, n=72) with untreated early rheumatoid arthritis (ueRA) and healthy controls (HC, male + female, n= 31). (a) OPLS-DA score scatter plot showing the separation of ueRA and HC based on T cell subsets proportions. (b) OPLS-DA column loading plot depicting the association between ueRA or HC (Y-variables) and T cell subset proportions (X-variables). X-variables represented by bars pointing in the same direction as ueRA are positively associated, whereas variables in the opposite direction are inversely related to ueRA. Asterisks indicate significant difference between ueRA and HC in Two-tailed Mann-Whitney U-test. *P ≤ 0.05, **P ≤ 0.01, ***P ≤ 0.001 and **** P≤ 0.0001.

## Slide 3
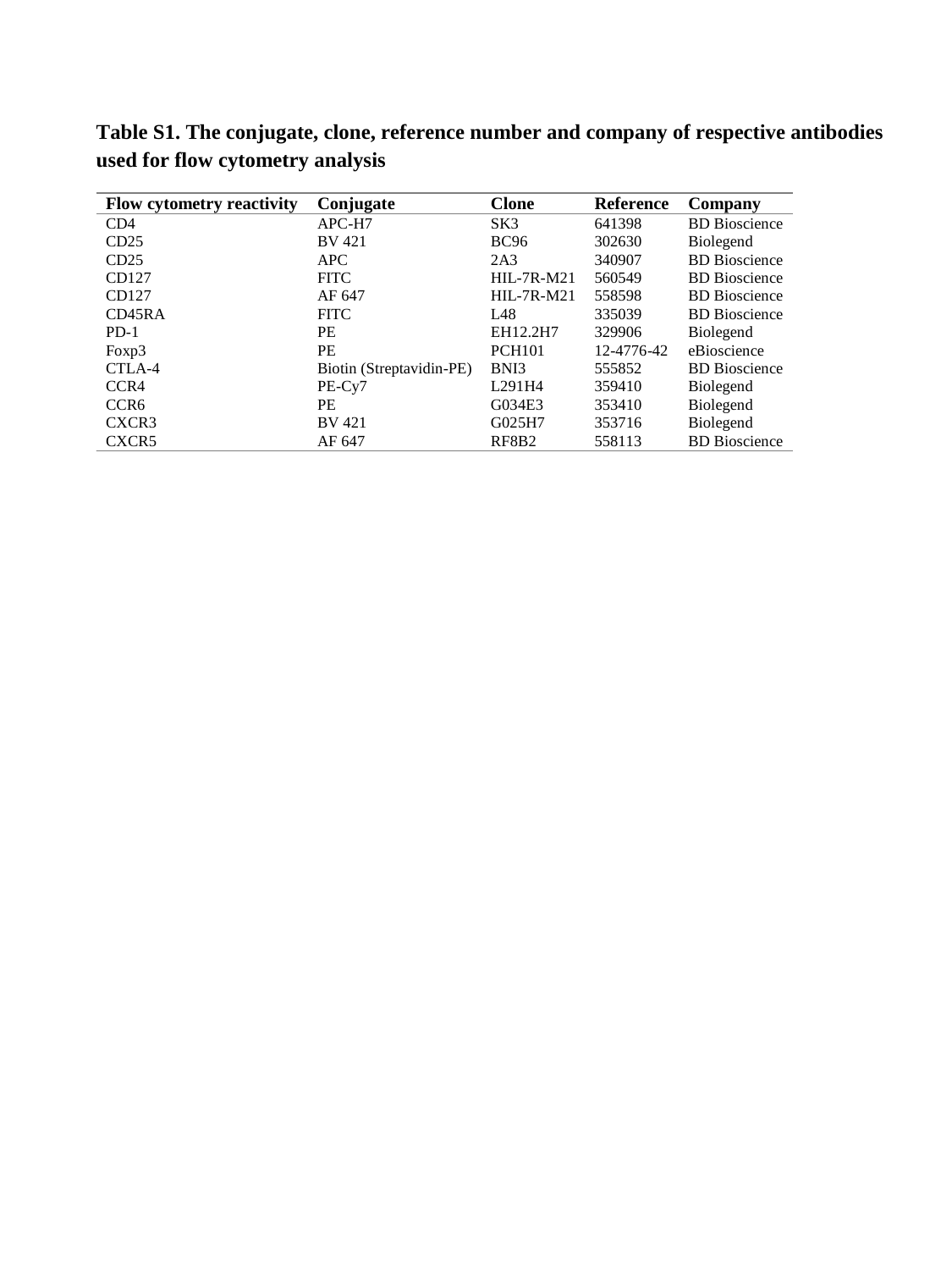

Supplement: Supplementary file 1 — Figure S1. Differences in the proportions of T cell subsets between untreated early RA and healthy controls. OPLS-DA scatter and loading plots resulting from multivariate factor analysis of ueRA patients (male + female) vs HC (male + female). Table S1. The conjugate, clone, reference number and company of respective antibodies used for flow cytometry analysis. List of antibodies used in flow cytometry analysis and the respective conjugate, clone, reference number and company for each of these. (PPTX 99 kb) [file 13075_2018_1648_MOESM1_ESM.pptx]
